# The potential of caproate (hexanoate) production using *Clostridium kluyveri* syntrophic cocultures with *Clostridium acetobutylicum* or *Clostridium saccharolyticum*

**DOI:** 10.3389/fbioe.2022.965614

**Published:** 2022-08-22

**Authors:** Jonathan K. Otten, Yin Zou, Eleftherios T. Papoutsakis

**Affiliations:** ^1^ Department of Chemical and Biomolecular Engineering, University of Delaware, Newark, DE, United States; ^2^ Delaware Biotechnology Institute, University of Delaware, Newark, DE, United States; ^3^ Department of Biological Sciences, University of Delaware, Newark, DE, United States

**Keywords:** coculture (co-culture), *Clostridium* kluyveri, *Clostridium saccharolyticum*, *Clostridium* acetobutylicum ATCC 824, chain elongation, caproate, hexanoate, *Lacrimispora saccharolytica*

## Abstract

Caproate (hexanoate) and other medium-chain fatty acids are valuable platform chemicals produced by processes utilizing petroleum or plant oil. *Clostridium kluyveri*, growing on short chain alcohols (notably ethanol) and carboxylic acids (such as acetate) is noted for its ability to perform chain elongation to produce 4- to 8-carbon carboxylates. *C. kluyveri* has been studied in monoculture and coculture conditions, which lead to relatively modest carboxylate titers after long fermentation times. To assess the biosynthetic potential of *C. kluyveri* for caproate production from sugars through coculture fermentations, in the absence of monoculture data in the literature suitable for our coculture experiments, we first explored *C. kluyveri* monocultures. Some monocultures achieved caproate titers of 150 to over 200 mM in 40–50 h with a production rate of 7.9 mM/h. Based on that data, we then explored two novel, syntrophic coculture partners for producing caproate from sugars: *Clostridium acetobutylicum* and *Clostridium saccharolyticum*. Neither species has been cocultured with *C. kluyveri* before, and both demonstrate promising results. Our experiments of *C. kluyveri* monocultures and *C. kluyveri*—*C. saccharolyticum* cocultures demonstrate exceptionally high caproate titers (145–200 mM), fast production rates (3.25–8.1 mM/h), and short fermentation times (18–45 h). These results represent the most caproate produced by a *C. kluyveri* coculture in the shortest known fermentation time. We also explored the possibility of heterologous cell fusion between the coculture pairs similar to the results seen previously in our group with *C. acetobutylicum* and *Clostridium ljungdahlii.* Fusion events were observed only in the *C. acetobutylicum*—*C. kluyveri* coculture pair, and we offer an explanation for the lack of fusion between *C. saccharolyticum* and *C. kluyveri*. This work supports the promise of coculture biotechnology for sustainable production of caproate and other platform chemicals.

## 1 Introduction

Caproic (hexanoic) acid, as well as other medium-chain fatty acids (MCFAs), are platform chemicals, fuel precursors, antimicrobial agents, plant growth promoters, lubricant precursors, and flavor additives (Zhang et al., 2019; San-Valero et al., 2020; Ghysels et al., 2021). Currently, they are produced from fossil fuels or through low-yield extractions from coconut or palm kernel oil. As the world gravitates towards more sustainable sources of chemicals, petroleum-based processes will have to be phased out. Palm oil production also carries substantial environmental concerns (Syahril et al., 2019). Biological production of caproic acid and other MCFAs creates a solution to these environmental concerns.

An important organism capable of chain elongation from less-valuable precursors into MCFAs is *Clostridium kluyveri* (*Ckl*), a spore-forming anaerobe that produces caproic (hexanoic) acid (Thauer et al., 1968; Zhang et al., 2019). *Ckl* uses the reverse beta-oxidation pathway to produce MCFAs with ethanol as an electron donor and acetate as an electron acceptor (Richter et al., 2016; San-Valero et al., 2019; Zhang et al., 2019; Ghysels et al., 2021). It also uses other short chain alcohols and carboxylic acids, including propionate to produce pentanoate (valerate) and heptanoate (Candry et al., 2020), and propanol and succinate to produce propionate, butyrate, pentanoate, and caproate (Kenealy & Waselefsky, 1985). *Ckl* grows best with an ethanol:acetate ratio of 3:1 or higher (Bornstein & Barker, 1948; Yin et al., 2017).

Caproate can be slowly converted with a low fractional conversion to the C8 caprylate, and thus trace amounts of caprylate can be formed (Gildemyn et al., 2017). However, due to its low solubility in water (0.0068 g/L), caprylic acid is difficult to measure. Caproate is an essential metabolite to enable the growth of *Ckl* on ethanol and acetate. The energy metabolism of *Ckl* has been extensively studied, and is now well understood (Seedorf et al., 2008). As shown in the following reactions (Seedorf et al., 2008), production of caproate through a coupled reaction network is essential to the energy metabolism of *Ckl*.

$$1\;ethanol+1\;H_{2}O\to 1\;acetate^{-}+1\;H^{+}+2\;H_{2}\Delta {\text{G}}^{\text{°}\prime }=\;+9\text{.}7\text{ kJ/mol}$$ [1]

$$4\;ethanol+4\;acetate^{-}\to 4\;butyrate+4\;H_{2}O\Delta {\text{G}}^{\text{°}\prime }=\;-38\text{.}6\text{ kJ/mol}$$ [2]

$$1\;ethanol+1\;butyrate\to 1\;caproate+1\;H_{2}O\Delta {\text{G}}^{\text{°}\prime }=\;-38\text{.}6\text{ kJ/mol}$$ [3]

The productivity of *Ckl* monocultures growing on ethanol and acetate remains low due to excessive fermentation times. For example, it can produce 83–110 mM of caproate in 38–72 h (Weimer & Stevenson, 2012; Candry et al., 2018; Ghysels et al., 2021). With expanded timescales of 150–200 h, production of 167–181 mM of caproate has been reported (San-Valero et al., 2019; San-Valero et al., 2020). Other strategies such as immobilization (Zhang et al., 2019) or additions of biochar or activated carbon (Ghysels et al., 2021) have improved caproate titers. Table 1 summarizes key literature reports.

**TABLE 1:** Caproate titers and production rates of *Ckl* monocultures.

Max caproate titer (mM)	Max ethanol titer (mM)	Hours	Caproate productivity^a^ (mM/h)	pH range	Special conditions	References
219	443	45	4.87	7	One replicate	This work
181	348	192	0.94	6.8	None	San-Valero et al. (2019)
173	485	45	3.84	7	None	This work
167	543	160	1.04	7.5	None	San-Valero et al. (2020)
124		120	1.04	8.1 to 6.1	Recycled biochar	Ghysels et al. (2021)
117	340	18	6.50	6.8	None	This work
110	700	72	1.53	5 to 9	None	Weimer and Stevenson, (2012)
96	343	38	2.53	8.2 to 7.4	None	Candry et al. (2018)
95	315	90	1.06	6.8 to 6.0	Biofilm reuse	Zhang et al. (2019)
94		116	0.81	6.8 to 6.0	Immobilization	Zhang et al. (2019)
91	275	55	1.65	8.1 to 6.6	Activated carbon	Ghysels et al. (2021)
87	265	55	1.58	8.1 to 6.2	Biochar	Ghysels et al. (2021)
83	295	55	1.51	8.1 to 6.2	None	Ghysels et al. (2021)
71		165	0.43	6.8 to 6.0	Ammonia supplementation	Zhang et al. (2019)
65	543	50	1.30	7.5	None	San-Valero et al. (2020)
52	350	42	1.24	5 to 9	None	Weimer and Stevenson, (2012)
35	240	48	0.73	start 6.5	None	Thauer et al. (1968)

a Calculated as the maximum total caproate titer (mM) divided by the fermentation time needed to reach that maximum (h).

Ethanol and acetate are expensive substrates for hexanoate production, so to lower substrate costs, cocultures of *Ckl* have been explored. Potential coculture partners such as *Clostridium acetobutylicum* can utilize a wide variety of sugar feedstocks, including all mono- and oligosaccharides derived from biomass. In nature, microbes live in complex communities, and, thus, it is not surprising that native or synthetic cocultures can improve the efficiency of substrate utilization, improve product yields, and broaden the metabolic space by producing products that cannot be formed by single species alone (Charubin & Papoutsakis, 2019; Cui et al., 2021; Diender et al., 2021). *Ckl* has been studied in coculture with gas-consuming acetogens such as *Clostridium ljungdahlii* (Richter et al., 2016) and the closely-related *C. autoethanogenum* (Diender et al., 2019; Benito-Vaquerizo et al., 2020), and with Methanogen 166 (Yan & Dong, 2018). *Ckl* has also been studied in the complex environment of anaerobic sludge (Zagrodnik et al., 2020) with mixed substrates of lactose, lactate, ethanol, and acetate; and with ruminal microflora and cellulolytic bacteria (Kenealy et al., 1995; Weimer et al., 2015) with cellulose or cellulosic biomass and supplemental ethanol as substrates. Syntrophic cocultures with *C. ljungdahlii* or *C. autoethanogenum* are noted for their growth on syngas (CO_2_/H_2_/CO) to transform waste gases into valuable chemicals. However, caproate production from these cocultures reached maximum titers of 7.5–11 mM (Richter et al., 2016; Diender et al., 2019), which are of little practical significance. Hexanol and octanol production, formed in these cocultures by the conversion of a fraction of the corresponding carboxylates, are likewise minimal. Cocultures with methanogens and with ruminal bacteria, or in anaerobic sludge, came closer to matching the product-formation performance of *Ckl* monocultures, but as summarized in Table 2, these cocultures display low caproate productivity: maximum titers of 90 mM and long fermentation times (>6–7 days).

**TABLE 2:** Caproate titers, production rates, and coculture partners of *Ckl* cocultures.

Max caproate titer (mM)	Max ethanol titer (mM)	Hours	Caproate productivity^a^ (mM/h)	pH range	Coculture species	Substrate & (special conditions)	References
161	135	136	1.18	7	*C. saccharolyticum*	Glucose	This work
120	75	41	2.93	7	*C. saccharolyticum*	Glucose	This work
65	150	456	0.14	7.5 to 5.5	Clostridia-rich anaerobic digester sludge	Mixed substrate of lactose, lactate, acetate, and ethanol	Zagrodnik et al. (2020)
54	150	168	0.32	7.5 to 5.5	Clostridia-rich anaerobic digester sludge	Mixed substrate of lactose, lactate, acetate, and ethanol	Zagrodnik et al. (2020)
53	225	48	1.09	6.8 to 5.7	Ruminal bacteria	Cellulosic biomass with supplemental ethanol	Weimer et al. (2015)
40	96	112	0.36	6.8	Ruminal bacteria	Cellulose with supplemental ethanol	Kenealy et al. (1995)
31	130	22	1.41	6.2	*C. acetobutylicum*	Glucose	This work
11	28	300	0.04	6	*C. ljungdahlii*	Syngas (gas stripping)	Richter et al. (2016)
8	45	168	0.04	6.2	*C. autoethanogenum*	Carbon monoxide with supplemental ethanol	Diender et al. (2019)

a Calculated as the maximum total caproate titer (mM) divided by the fermentation time needed to reach that maximum (h).

Our goal in this work is to explore new syntrophic coculture partners for *Ckl* that will maximize caproate production with shorter fermentation times. *C. acetobutylicum* (*Cac*) and *C. saccharolyticum* (*Csh*) were our two species of choice. In the published literature, neither has been reported in coculture with *Ckl.* Both are capable of good ethanol and acetate production to enable good syntrophic coculture productivity. In syntrophy, one organism depends for survival on the metabolic products of one or multiple organisms (Charubin et al., 2020). Here *Ckl* is the dependent partner fed by an organism (feeder partner) grown on sugars that produces ethanol and acetate to feed *Ckl*. *Cac* is a model organism for acetone-butanol-ethanol fermentation. We have previously reported the engineered strain *Cac* 824 (pCASAAD), carrying plasmid pCASAAD, produces the highest amount of ethanol observed in a *Cac* fermentation (Sillers et al., 2009). pCASAAD overexpresses the native fusion alcohol/aldehyde dehydrogenase enzyme, which catalyzes both ethanol and butanol formation. It also expresses an antisense RNA molecule, which targets the transcript of the CoA-transferase to result in reduced acetone production. The high ethanol titers (over 300 mM for *Cac*-pCASAAD) and the 3:1 ethanol:acetate ratio of its two metabolic products make *Cac* and *Cac*-pCASAAD good candidates as *Ckl* syntrophic coculture partners.

As *Cac* produces several products (notably butanol, acetone, and butyrate) in addition to ethanol and acetate, we also examined an alternate feeder organism with more favorable profile of metabolites. *Csh*, an ethanologenic anaerobe that produces ethanol, acetate, and lactate (Khan and Murray, 1982), was chosen because of its ability to produce high concentrations of ethanol and acetate in the 3:1 ratio and at neutral pH, both of which are favorable for *Ckl* growth (Richter et al., 2016). *Csh* has been studied in coculture with cellulolytic organisms (Khan and Murray, 1982; Murray, 1986), but not with organisms that produce MCFAs. Its growth at neutral pH indicates good compatibility with *Ckl*.

In designing coculture systems for metabolite production, one needs to understand the capabilities of each organism in terms of substrate utilization (rates and tolerance) and metabolite production (rates, tolerance, and titers) in the medium one intends to utilize for the coculture. As there are no satisfactory such data for *Ckl* in the literature and certainly none for growth media suitable for our cocultures, we first examined the metabolic capabilities of *Ckl* in monocultures: rates of growth, substrate utilization, and metabolite production. This was followed by coculture experiments. We demonstrate the most productive published *Ckl* monocultures. Cocultures of *Ckl* with *Cac* produced more caproate than *C. ljungdahlii* or *C. autoethanogenum* cocultures. Cocultures of *Ckl* with *Csh* demonstrated record-high and record-fast productions of caproate. We also found that heterologous cell fusion between *Ckl* and *Cac* in coculture similar to recently demonstrated fusions between *Cac* and *Clj* (Charubin and Papoutsakis, 2019; Charubin et al., 2020; Charubin et al., 2021). Such fusion events are of interest for further exploration for the development of hybrid cells (Charubin et al., 2020).

## 2 Materials and methods

### 2.1 Microorganisms and growth media

*C. acetobutylicum* (ATCC 824, *Cac*) and *C. kluyveri* (ATCC 8527, *Ckl*) monocultures and cocultures were grown in a growth medium Turbo CGM (Charubin and Papoutsakis, 2019) with the following modifications. Across all culture conditions, the potassium phosphate buffer addition was doubled to 20 ml/L. This buffer consists of 100 g/L of KH_2_PO_4_ and 125 g/L of K_2_HPO_4_ adjusted to a pH of 6.8. *Cac* monocultures were grown in the following medium (termed T-CGM-G): high-buffer Turbo CGM with 80 g/L glucose. *Ckl* monocultures used the following medium (termed T-CGM-NA): high-buffer Turbo CGM with 0 g/L glucose, 8.0 g/L sodium acetate, 15.8 g/L ethanol, 2.5 g/L sodium bicarbonate, and 0.3 g/L L-cysteine HCl. Cocultures of *Cac-Ckl* were grown in the following medium (termed T-CGM-CC): high-buffer Turbo CGM with 40–80 g/L glucose, 2.5 g/L sodium bicarbonate, and 0.3 g/L L-cysteine HCl. *C. saccharolyticum* WM1 (ATCC 35040, *Csh*) was grown in monocultures and cocultures in T-CGM-CC with 40 g/L of glucose. To prepare bioreactors for fermentation experiments, they were autoclaved with T-CGM-BR medium, which is T-CGM-CC before the addition of the phosphate buffer, bicarbonate, L-cysteine HCL, glucose, and Wolfe’s vitamins (Charubin & Papoutsakis, 2019). Sterile solutions of these chemicals were added after the medium cooled off to form T-CGM-CC.

### 2.2 Culture of individual microorganisms to prepare inocula for monocultures and cocultures

To begin a *Ckl* culture, a 1.3 ml frozen stock (20% [v/v] glycerol, stored at -80°C) was inoculated in a 100 ml GL-45 media bottle containing 80 ml of T-CGM-NA and grown at 37°C in the incubator of an anaerobic chamber (Thermo Forma 1025) containing an atmosphere of 85% N_2_, 10% CO_2_, and 5% H_2_. The lids of the media bottles were slightly open to allow for gas exchange and the release of excess pressure. After 3 days, once the OD_600_ (Optical Density at 600 nm) reached ∼0.5, the initial culture was passaged with a 10% inoculum to fresh T-CGM-NA. This resulting culture can be passaged as needed and cultured under identical conditions to generate additional cells. To prepare inocula for mono- and coculture fermentations, *Ckl* was concentrated with anaerobic centrifugation in multiple 50 ml plastic conical tubes at 4,000 rpm and room temperature for 8 min. The supernatant was then discarded, and the pellets were resuspended in fresh media. 3–5x concentration of cells was employed.

*Csh* frozen stocks (1.3 ml volume preserved in 20% [v/v] glycerol, stored at −80°C) were inoculated in 100 ml GL-45 media bottles containing 80 ml of T-CGM-CC in the same conditions as the *Ckl* frozen stocks overnight. The next morning, they were suitable for passaging into fresh T-CGM-CC at 10% inoculum to allow for growth to prepare for coculture conditions.

For *Cac*, a streak of a frozen stock (20% [v/v] glycerol, stored at -80°C) was applied onto 2xYTG plates and incubated anaerobically at 37°C for 3–4 days to allow for spore formation. A single colony was used to inoculate 10 ml of T-CGM-G that was heat shocked at 80°C for 10 min. This culture was then incubated at 37°C for 12 h to allow cells to reach mid-exponential phase. The cells could then be passaged at 10% (v/v) into fresh media to generate more cells. 8 h after the start of each passage, sterile 3M NaOH was used to raise the pH of the culture to 5.5-6.5 to prevent cell death and transition the *Cac* to solventogenesis. For 824 (pCASAAD), erythromycin was added to the plates at 40 μg/ml and to the liquid T-CGM-G medium at 100 μg/mL as specified (Sillers et al., 2009).

### 2.3 Growth of *Ckl* monocultures in serum bottles without pH control

To begin a monoculture fermentation of *Ckl*, cells were concentrated to an initial target OD_600_ of 1. The cells were then resuspended and inoculated in 100 ml GL-45 media bottles containing fresh T-CGM-NA as in Section 2.2.

### 2.4 Growth of *Cac* monocultures and cocultures in serum bottles without pH control

To begin a monoculture fermentation of *Cac*, a 10% inoculum was used (Section 2.2), which translated to an initial OD_600_ of roughly 0.5. Exponential-phase *Cac*, T-CGM-G, and appropriate antibiotics were utilized. The incubator temperature, gas composition, and 100 ml GL-45 media bottles used for the initial culturing of the organisms were also used to perform chambered mono- and coculture fermentations.

In coculture fermentations, *Ckl* was added after *Cac* acidogenesis was stopped *via* the addition of 3.0 M NaOH, around 10 h. Because *Ckl* grows more slowly than *Cac*, a ratio of R ≈ 4 was chosen for inoculation through previous lab experience with *Clostridium* cocultures (Charubin & Papoutsakis, 2019) based on the relative growth rate of the cells. R is defined as the ratios of cell concentration between *Ckl* and *Cac* or *Csh* based on OD_600_. Cocultures used T-CGM-CC and the same unpressurised bottles and chamber conditions as described in Section 2.2.

### 2.5 Construction and use of 150-ml bioreactors with pH control

For pH-controlled fermentations, small bioreactors were constructed (Supplementary Figure S1). This bioreactor setup uses spinner flasks (Chemglass CLS-1400-100). An active volume of 150 ml was used to cover the pH probe, which was inserted into one of the arms through a rubber grommet (McMaster-Carr 1061T17) into the open aperture of a GL-32 cap. The main reactor lid was solid without a central stirring axle, as a free-spinning stir bar was used instead. The other GL-32 opening held a solvent delivery cap with 4 ¼-28 threaded ports (Cole-Parmer EW-12018-53). These ports were used for sampling, base injection, gas input, and exhaust output. Each vessel’s pH was individually controlled with 1.8 M sterile NaOH by pH controllers (Bluelab pH Controller Connect). Each vessel had individually controlled nitrogen sparging to allow the initial creation of an anaerobic environment, with adjustable flow rates and a pressure of 0.5 psi. All exhaust bubbled through a water-filled bottle to ensure that an anerobic environment was maintained. 2-3 glass vessels were placed in fiberglass bins set atop individual stir plates, which turned the stir bars in each fermentation vessel. Water was used to fill the bins around the exterior of each vessel, and the water temperature was maintained at 37°C with sous vide circulators.

To ensure sterility, all vessels were autoclaved with 150 ml T-CGM-BR before the addition of the phosphate buffer, L-cysteine HCl, bicarbonate buffer, glucose, or Wolfe’s vitamins. After the vessels were removed from the autoclave and cooled, the remaining media supplements were added to create T-CGM-CC in the bioreactors. The pH probes were calibrated and sterilized with ethanol before being added to the vessels, and then nitrogen was sparged through the vessels for several hours as the temperature of the exterior water bins was increased to 37°C. The vessels were then ready for inoculation with syringes of concentrated cells prepared in the anaerobic chamber. For *Ckl* monocultures, cells were added immediately to a target OD_600_ of 1. For *Csh*/*Ckl* cocultures, *Csh* was added immediately to a target OD_600_ of 0.2. *Ckl* was added an hour later at a target ratio (*Ckl/Csh*) of *R* = 4.

### 2.6 Metabolite analysis

All cultures were sampled every 6–12 h until fermentation ended. The OD_600_ and pH were recorded, with 1/10 and 1/20 dilutions used for the OD_600_ as applicable so that the spectrophotometer never read a value of over 0.5 to reduce error. The metabolite composition of the media was analyzed with high-performance liquid chromatography as described (Carlson and Papoutsakis, 2017). The OD_600_ of the monocultures of *Ckl* were correlated with the dry weight of the cells by centrifuging 100 ml of cell-containing media at 5,000 rpm for 15 min and then drying the samples in pre-massed weigh boats at 80°C and subtracting the mass of the same volumes of dried cell-free media.

### 2.7 Fluorescent cell staining for microscopy and flow cytometry analysis

*Ckl* cells were stained with CellTracker^TM^ Green CMFDA (CTG) (Grego et al., 2013). *Cac*-pCASAAD and *Csh* cells were stained with CellTracker^TM^ Deep Red (CTDR) (Zhou et al., 2016). To prepare these dyes, the CTG is centrifuged then mixed with 80 μL of DMSO, and CTDR is centrifuged and mixed with 20 μL of DMSO. This creates a 1000x solution for staining cell culture media, which is anaerobically incubated for 30 min before centrifugation in 50 ml conical tubes for 8 min at 4,000 rpm. After a wash and resuspension in the T-CGM-CC, the stained cells are ready for culture.

Stained cells can be analyzed with a BD FACS Aria II flow cytometer immediately or at subsequent timepoints as described (Charubin et al., 2020)*.* To prepare for microscopy, 8-well μ-Slides with ibiTreat (ibidi) were incubated with 200 μL of 0.1% (w/v) poly-L-lysine overnight. Slides were then washed with sterile water and seeded with 200 μL of culture media that had been adjusted to an OD_600_ of 0.3. After 30 min of incubation in the anaerobic chamber incubator, wells were washed in three series of three washes with sterile PBS. After the final wash and aspiration, wells were covered with three drops of ProLong Glass (Invitrogen^TM^), which enables long-term storage and protects from photobleaching. The slides were then observed with a Zeiss LSM-900 with Airyscan. Z-stacks were taken of each cell of interest to examine heterologous cell interactions as described (Charubin et al., 2020).

## 3 Results and discussion

### 3.1 *C. kluyveri (Ckl)* monocultures demonstrate the potential of the organism for efficient and fast caproate production

In order to design the targeted coculture experiments, we first examined the potential and metabolic characteristics of *Ckl* to produce caproate from ethanol and acetate in shorter-duration fermentations in media suitable for subsequent cocultures with *Cac* and *Csh*. We aimed to reduce the fermentation time and to explore a culture pH range that will be compatible with two prospective syntrophic coculture partners. Literature data (Table 1) show that the highest caproate titers are achieved after long fermentation times. Peak caproate titers of 96 mM were reported after 38 h of fermentation in a pH range of 8.2–7.4 (Candry et al., 2018). The highest caproate titers from *Ckl* were reported as 110 mM in 72 h from a bovine-derived strain with 700 mM of ethanol (Weimer and Stevenson, 2012), and 124 mM in 120 h when biochar is added to the fermentation media (Ghysels et al., 2021). As *Ckl* grows slowly, and batch *Ckl* fermentations typically have a maximum OD_600_ below 0.8, to reduce culture time, we hypothesized that a concentrated *Ckl* inoculum might prove beneficial, although there is no assurance that higher starting cell densities will lead to higher rates of metabolite production and titers, as several other factors are known to affect those. Concentrated inocula are routinely used in industrial practice. Thus, we tested the impact of centrifugation (for cell concentration) and passaging on *Ckl* growth. When the same amount of an initial culture of *Ckl* was either directly passaged, centrifuged and passaged, or centrifuged and passaged along with the supernatant, no major differences in OD_600_, pH, or metabolite profiles were observed (data not shown), so we conclude that the centrifugation process does not harm the *Ckl* cells.

**FIGURE 1:**
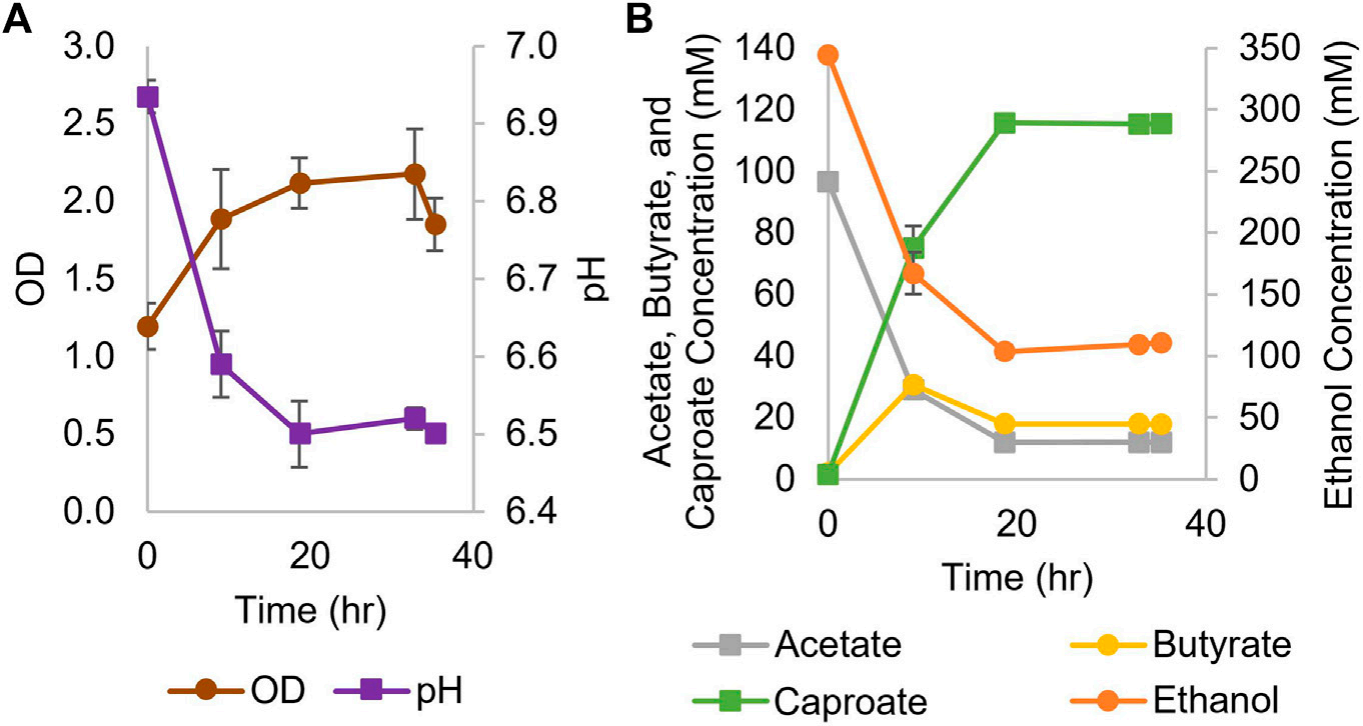
Growth and metabolite profiles of *Ckl* cultures for caproate production from ethanol and acetate. **(A)**: OD and pH of cultures in serum bottles (*n* = 2 biological replicates). OD_600_ can be converted to mgCDW/mL by multiplying the OD_600_ by 0.43. **(B)**: Metabolite concentrations. Note the separate axis for ethanol concentration.

While *Ckl* has most often been cultured in the 7.4–8.2 pH range (Weimer and Stevenson, 2012; Candry et al., 2018), it can also be grown at culture pH between 6 and 7 (Zhang et al., 2019; Ghysels et al., 2021). Thus, we performed a screening experiment at seven different pH values, from 5-6.25, as well as at 7.0, to determine if *Ckl* could grow in a pH range that was compatible with *Cac*. *Ckl* grew to the highest OD_600_ and produced the most caproate at a starting pH of 7; no growth was observed below pH 5.75, and at pH settings lower than 7, less than half as much caproate was produced than at pH 7 (data not shown). Based on these findings, we concluded that the cocultures required at least some periods of growth at pH 7 for caproate production to proceed. Our monoculture experiments employed a concentrated inoculum *via* centrifugation and started at pH 7 aiming to achieve fast caproate production. Figure 1 shows the results of two biological replicates of a *Ckl* monoculture in serum bottles, whereby cells were concentrated by centrifugation and resuspended in fresh T-CGM-NA medium. Culture medium was buffered with both bicarbonate and potassium phosphate, but no base was added during the fermentation. The starting OD_600_ was about 1.2 and rose to 2.2 within 36 h. Based on our found correlation factor of 0.43, this represents 0.51 to 0.94 mgCDW/mL. pH dropped to 6.5 from a starting pH of 6.93. In this set of fermentations, with a starting 3:1 M ratio of ethanol to acetate, *Ckl* produced 115 mM of caproate in less than 20 h at a rate of over 8.1 mM/h or 5.1 mM/h-OD_600_ between hours 0 and 9. Minimal butyrate production with some butyrate uptake was observed. Caproate was the main product, with an almost homo-caproate fermentation. It is possible that caproate production could have continued if ethanol and acetate concentrations were maintained at higher levels, the pH was controlled, or if product inhibition did not occur.

**FIGURE 2:**
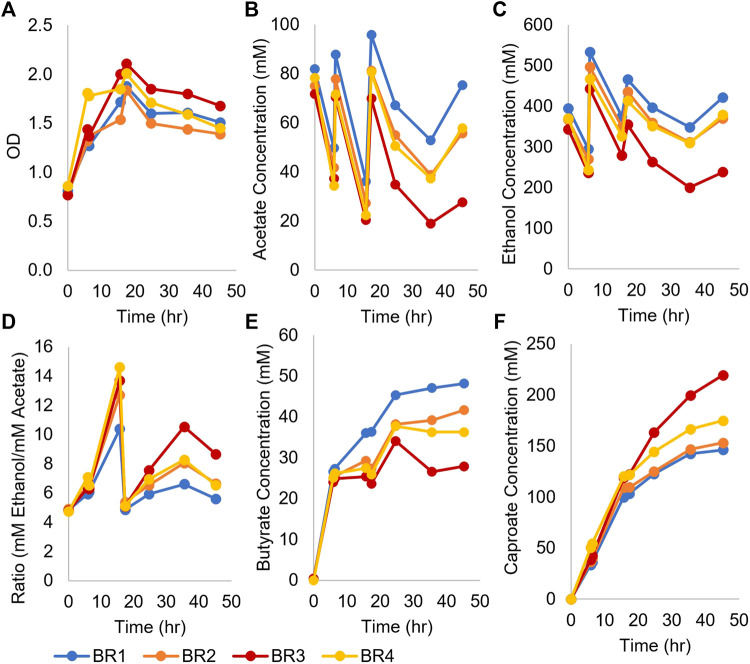
Growth and metabolite profiles of four *Ckl* cultures in pH-controlled bioreactors. The OD_600_ values remained high throughout the fermentation. OD_600_ can be converted to mgCDW/mL by multiplying the OD_600_ by 0.43. **(A)**. Ethanol and acetate were added twice at *t* = 6 and *t* = 16 h **(B,C)**. The ethanol/acetate ratio remained >4 throughout the fermentation **(D)** and butyrate concentrations were far lower than caproate concentrations **(E)**. Note that the culture with the lowest ethanol and butyrate concentrations had the highest caproate titer **(F)**, 219 mM. The positive slopes in **(F)** indicate continued caproate production.

To determine if higher production of caproate was possible, four monoculture fermentations inoculated with concentrated *Ckl* were performed in pH-controlled bioreactors using T-CGM-NA (Figure 2). pH was controlled at 7, and cells grew from their initial OD_600_ of 0.8 to a peak of ∼2 before stabilizing at an OD_600_ of ∼1.5 (Figure 2A). Ethanol was added twice to maintain its titer above 200 mM (Figure 2C). With acetate added twice as well, the ethanol/acetate ratio remained above 4 (Figure 2D). With these conditions, over 150 mM of caproate was produced in 45 h (Figure 2F), which exceeds the caproate production seen in Figure 1B. One replicate performed even better, producing 219 mM of caproate. This replicate, BR3, consumed the most ethanol and acetate and produced the least butyrate. This caproate titer is the highest ever reported for a *Ckl* monoculture (Table 1), and critically, it was obtained without any *in situ* removal of caproate. Its peak production rates mirrored the rates of the fermentations without pH control (Figure 1B), with 7.9 mM/h or 5.3 mM/h-OD_600_ of caproate produced. We conclude that the presence of high titers of ethanol (over 200 mM) and a neutral pH are beneficial in caproate production. We also note the positive slope to caproate formation in Figure 2F at 48 h, indicative of the continuous capacity of the organism to produce caproate even after significant caproate accumulation, thus demonstrating a good tolerance to caproate toxicity.

We conclude that a concentrated inoculum dramatically decreases fermentation time and improves the caproate production of *Ckl* by enabling a much higher species ratio of the slow-growing *Ckl*. Published data reported fermentation times of 2–3 days to produce over 100 mM of caproate, utilizing either high pH values, twice higher ethanol concentrations, or biochar (Weimer & Stevenson, 2012; Candry et al., 2018; Ghysels et al., 2021). Our data argue for an optimized process using adapted concentrated *Ckl* inocula for fast production of caproate.

### 3.2 *C. acetobutylicum (Cac)* and *Ckl* cocultures demonstrate caproate production from sugars under syntrophic conditions

**FIGURE 3:**
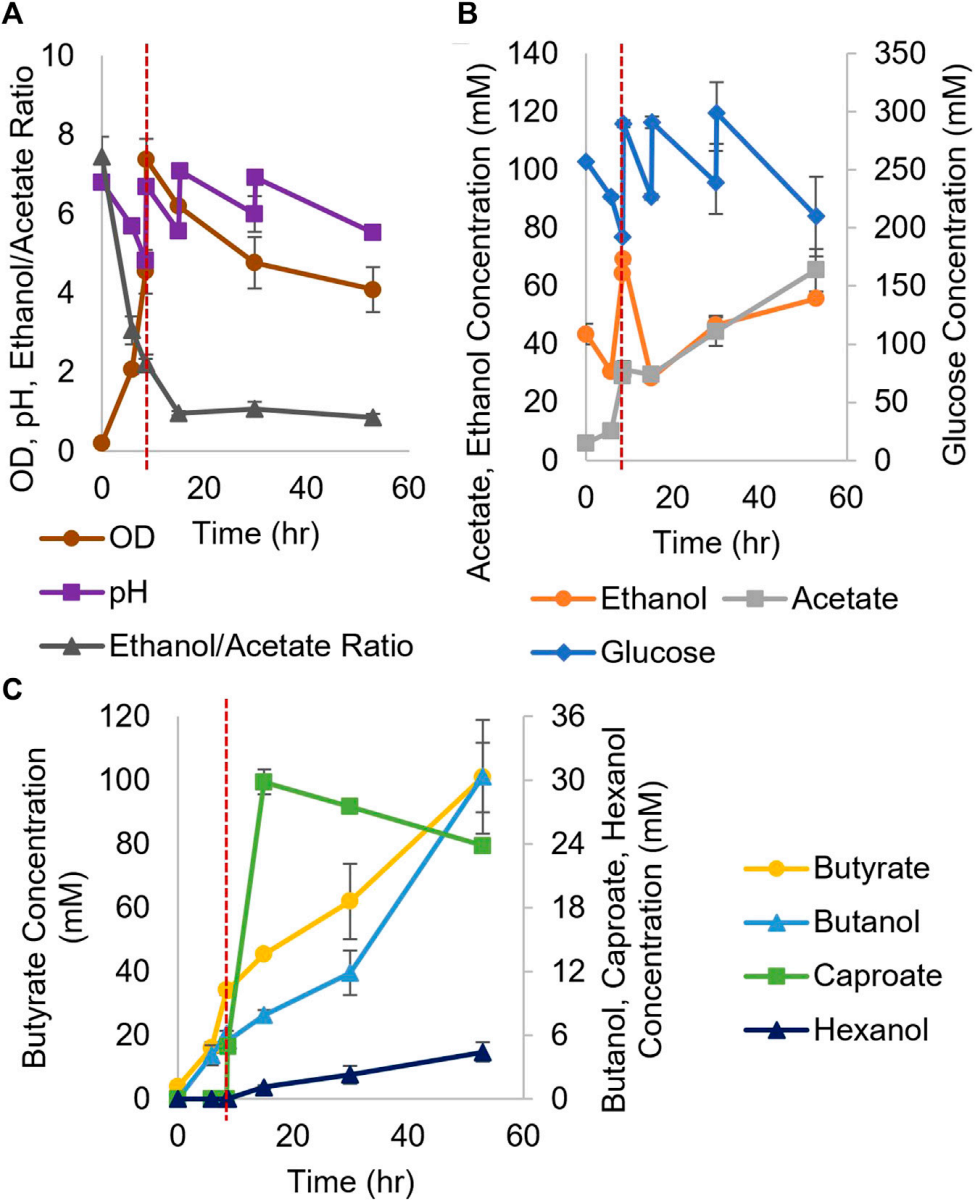
Growth and metabolite profiles of WT *Cac*/*Ckl* syntrophic cocultures. The pH was adjusted three times **(A)** (*n* = 3 biological replicates). In **(B)**, glucose was added three times (note the second axis) and ethanol titers remained low throughout the fermentation. 4- to 6-C product profiles are shown in **(C)** (note the second axis). Caproate was produced quickly, reaching its maximum titer at 20 h. The red dashed vertical line indicates *Ckl* inoculation.

We first wanted to explore the benefits of a coculture between *Ckl* and *Cac*. *Cac* can utilize a broad spectrum of biomass derived sugars: All 5 and 6-C sugars, oligosaccharides, and xylans (Papoutsakis, 2008; Tracy et al., 2012; Charubin et al., 2018). Thus, it would be beneficial to enable caproate production from such biomass-derived substrates. There are no literature reports of *Cac* and *Ckl* cocultures. Among other metabolites, *Cac* produces ethanol and acetate that *Ckl* needs for growth (Sillers et al., 2009; Lee et al., 2012). We also hypothesized that *Cac* will be able to reduce the carboxylic acids produced by *Ckl* into their respective alcohols. We tested three biological replicates of batch *Cac* and *Ckl* cocultures to assess their potential for caproate production (Figure 3). Nine hours into a *Cac* fermentation, more glucose and base (for pH control) were added just prior to inoculating the *Cac* culture with concentrated *Ckl* cells. Two more manual additions of sodium hydroxide (for pH control) and glucose were carried out at 15 and 30 h. The glucose additions were to ensure that *Cac* would not exhaust its feedstock. Because of autolysin formation at pH values at and above 6 (Croux et al., 1992), *Cac* began to lyse, and as expected this resulted in decreasing cell densities.

Both caproate (30 mM) and hexanol (5 mM) were produced (Figure 3C), which validates this novel syntrophic coculture. *Ckl* utilizes ethanol and acetate (and possibly butyrate) produced by *Cac*, while *Cac* converts caproate produced by *Ckl* into hexanol, as no hexanol production is possible in *Ckl* monocultures. Notable is the exceptional rate of caproate production (4.2 mM/h or 0.9 mM/h-OD_600_) between hours 9 and 16. The performance of the coculture in terms of caproate production deteriorates once the ethanol to acetate ratio falls below 2. This identifies the need for improvements. As WT *Cac* does not produce ethanol fast enough, a strain producing ethanol at higher rates will need to be identified or designed. This is pursued in the next two sections.

### 3.3 Exploring the use of *Cac* strain 824 (pCASAAD) for syntrophic cocultures with *Ckl* and the possibility of heterologous cell fusion between *Cac* and *Ckl*

Given the need for higher ethanol concentrations to support the growth of *Ckl*, we explored the use of the 824 (pCASAAD) *Cac* strain (Sillers et al., 2009) which produces much higher levels of ethanol compared to the parent WT *Cac* strain. We first tested the performance of 824 (pCASAAD) monocultures in chambered bottles (Supplementary Figure S2), the chosen setting for the cocultures. An OD_600_ of 9.5 was reached at 22 h, and another 24 h of fermentation further increased metabolite production. pH was adjusted twice with manual additions of base. 160 mM of ethanol was produced in 48 h, which far exceeds ethanol production by WT *Cac*. 50 mM of acetate was produced, which forms a desirable 3:1 ratio with ethanol. Still the major product is butanol, which reached 220 mM, and this may be an issue with the coculture due to the toxic effect of butanol.

**FIGURE 4:**
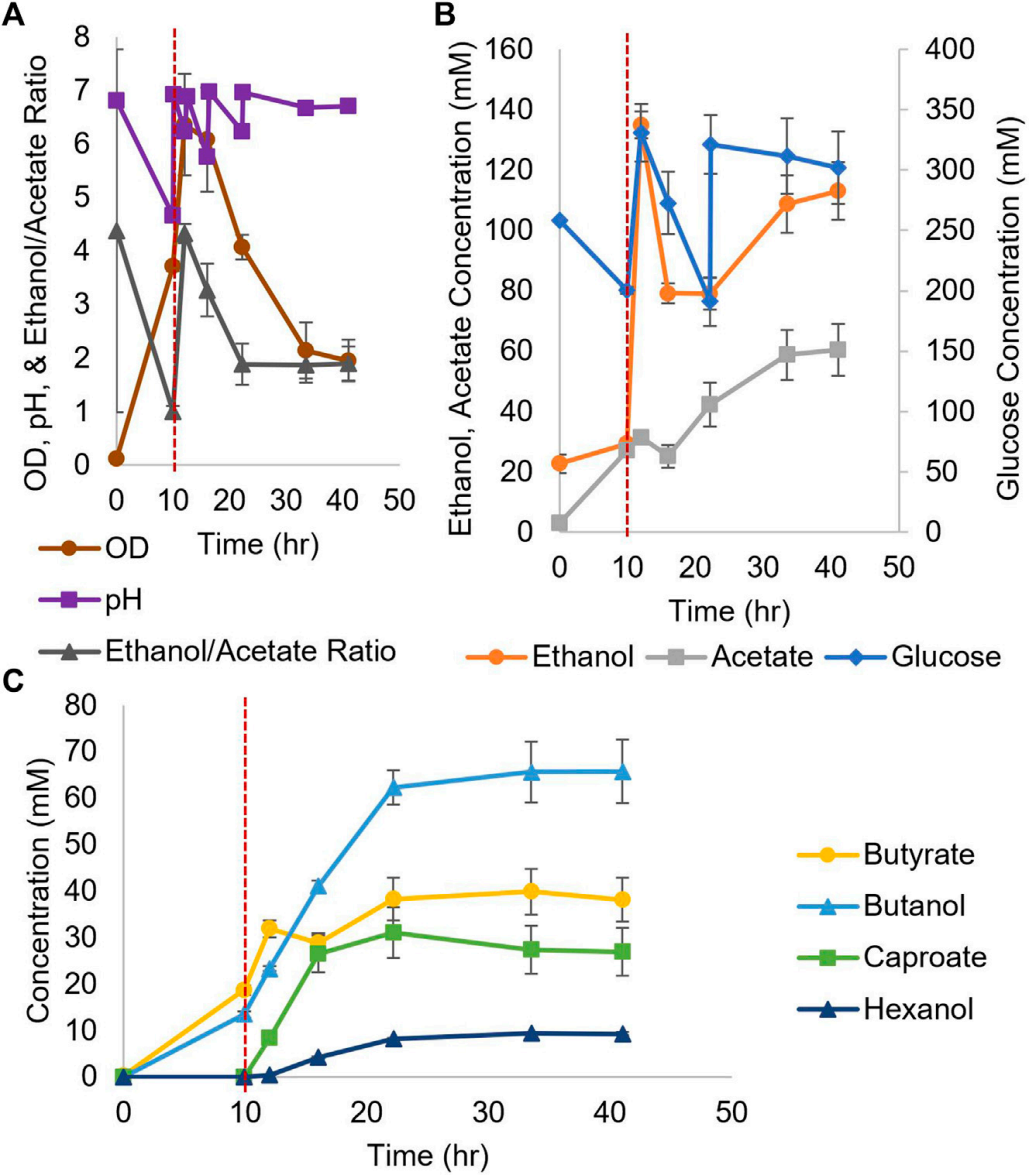
Growth and metabolite profiles of *Cac*(pCASAAD)/*Ckl* cocultures. **(A)** The profiles of OD_600_, pH, and the ethanol/acetate ratio (*n* = 3 biological replicates). The pH was raised three times to keep it largely over 6, and as a result OD_600_ of the coculture dropped quickly apparently due to autolysin production by *Cac* as discussed in the text. **(B)** The glucose profile and consumption (glucose was added twice; note the second axis) and the profiles of ethanol and acetate produced by *Cac*. Profiles of 4- and 6-C products are shown in **(C)**. Caproate was produced quickly, reaching its maximum value in about 20 hours. The red dashed vertical line indicates *Ckl* inoculation.

We thus carried out *Ckl* cocultures with 824 (pCASAAD) (Figure 4). After 10 h of 824 (pCASAAD) culture, when the pH dropped below 5 and OD_600_ reached almost 4, the pH was raised to 7.0 and a concentrated *Ckl* inoculum and glucose were added (Figure 4A). The pH was raised to 7.0 three subsequent times, and additional glucose was added at 22 h.

This fermentation produced 30 mM of caproate with a maximum production rate of 4.3 mM/h or 0.9 mM/h-OD_600_ and, notably, 10 mM of hexanol with a maximum production rate of 1 mM/h (Figure 4C). While the ethanol/acetate ratio remains close to 2 (Figure 4B), caproate production ceased by 22 h, although trace amounts of hexanol continued to be produced. The engineered 824 (pCASAAD) doubled the hexanol production relative to the WT *Cac* coculture. The lower ethanol and caproate titers relative to the monocultures and the drop in coculture OD (Figure 4A) indicate that the expected pH-mediated *Cac* autolysin release (Croux et al., 1992) continues to impact the viability and productivity of *Cac* cells. While excess ethanol and acetate remained in a ratio greater than 1, which should lead to more caproate production by *Ckl*, the quickly-falling OD lends support to the hypothesis that autolysin formation is impairing the performance of *Ckl* and the coculture at large.

To tune this coculture further and eliminate butanol and hexanol production, the *adhE1* and *adhE2* genes (Yoo et al., 2016) in *Cac* could be knocked out. High butyrate titers are seen in both Figures 3, 4. Butyrate is an intermediate chemical towards caproate production as is well established (Seedorf et al., 2008). With process optimization, including a strategy to increase ethanol concentrations, butyrate can be converted to caproate based on the kinetics and thermodynamics of well-established reactions (Seedorf et al., 2008), notably reaction [3] above. Ethanol can be sourced either through additions of biologically-produced exogenous ethanol or ethanol produced in coculture. The need for more ethanol and fewer side products led us to consider *Csh* as a coculture partner, which eliminates all butanol production. We believe our ideas and data set the foundation for process development and process optimization to realize an industrial production of caproate from sugars.

**FIGURE 5:**
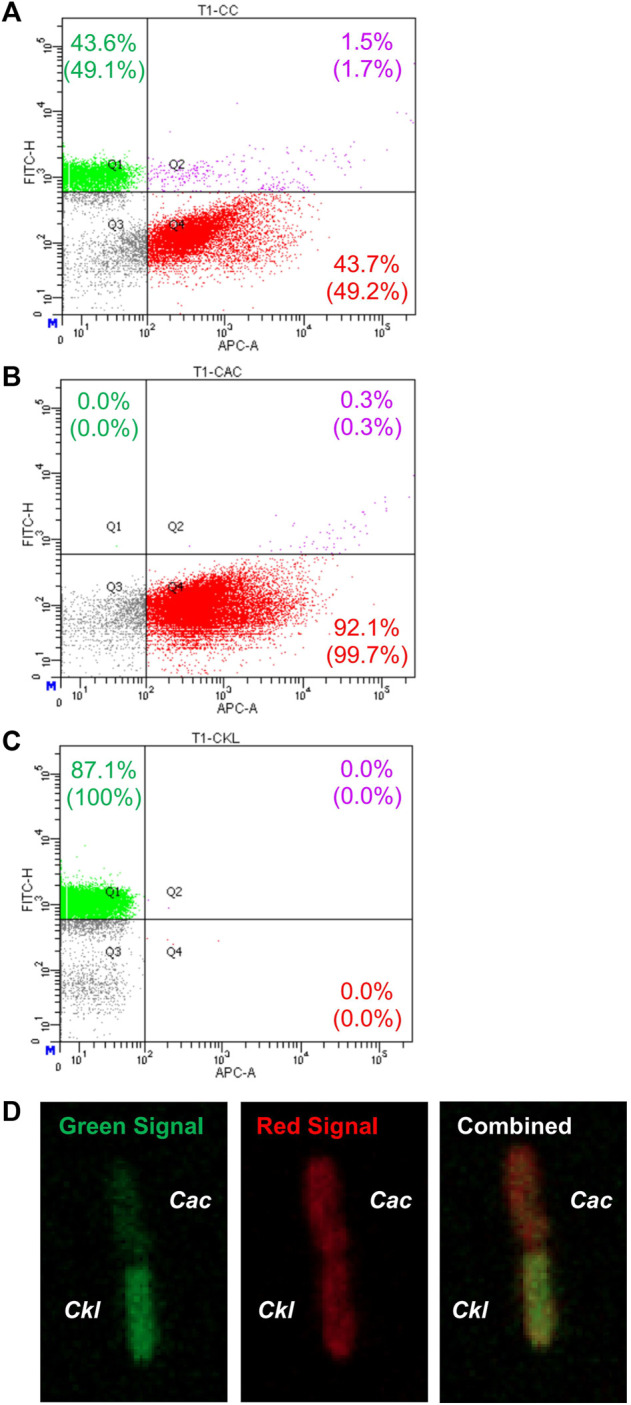
Evidence of heterologous cell fusion between *Cac*(pCASAAD) and *Ckl*. **(A–C)** shows representative flow-cytometric profiles of a *Cac*(pCASAAD)/*Ckl* coculture using stained cells. *Cac* cells were stained with CTDR, which expresses red fluorescence and can be seen in the APC channel. *Ckl* cells were stained with CTG, which expresses green fluorescence and can be seen in the FITC channel. **(A)** is the coculture, **(B)** is the *Cac* monoculture, and **(C)** is the *Ckl* monoculture. The percentages without parentheses represent the total number of cells, and the percentages within parentheses represent the normalized values of only fluorescent cells. Note the group of double-positive cells in Q2 of the coculture panel. **(D)** shows a confocal-microscopy image of a red-dyed *Cac* cell and a green-dyed *Ckl* cell. Both cells exhibit red and green signal, but the combined image on the right shows that the cells maintain their original dyed colors more strongly.

Nevertheless, in view of these data and given that syntrophic WT *Cac-Clj* cocultures led to heterologous cell fusion (Charubin et al., 2020), we examined this possibility for heterologous cell fusion in the *Cac-Ckl* coculture. We used both flow cytometry and fluorescence microscopy (Charubin et al., 2020). *Cac* 824 (pCASAAD) cells were stained with the CTDR dye and *Ckl* cells were stained with the CTG dye and cocultured in T-CGM-CC. Flow cytometry showed that a good population of (CTDR, CTG) double-positive cells at 3.3 h of the coculture (Figure 5A) thus suggesting that the cells are exchanging proteins as a result of heterologous cell fusion. These double cells are not seen in either monoculture (Figures 5B,C). To verify the putative heterologous cell-fusion events, we examined the cells using confocal microscopy (Figure 5D). Heterologously fusing cells display the same pole-to-pole contact that was observed in the detailed documentation of *Cac-Clj* heterologous cell fusion (Charubin et al., 2020), whereby both cells display both fluorescent signals due to exchange of labeled cellular proteins, but with different intensities: the green fluorescent intensity is higher in the CTG dyed *Ckl* cells and the red fluorescence higher in the CTDR dyed *Cac* cells. We conclude that *Cac* and *Ckl* form heterologous cell fusions similar to the documented *Cac-Clj* fusions. With these data in mind, it is possible to pursue the evolution of these cocultures by subculturing to examine if they may lead to novel stable phenotypes of hybrid cells as reported for the *Cac-Clj* pair (Charubin et al., 2021).

### 3.4 *C. saccharolyticum* is an effective coculture partner for *Ckl*, dramatically improving coculture-based caproate production from sugars

Our data show that *Cac* and *Ckl* could form successful partners in novel syntrophic cocultures, but their performance was hindered by fundamental pH incompatibilities, suboptimal rates of ethanol and acetate production by *Cac*, and production of *Cac* metabolites, such as butanol, that not only may reduce caproate selectivity, but also likely inhibit *Ckl* growth. *C. saccharolyticum* (*Csh*) (now reclassified as a *Clostridium* class, but genus *Lacrimispora* organism, *Lacrimispora saccharolytica*) (Haas & Blanchard, 2020), which produces mainly ethanol, acetate, and lactate at high titers (Murray et al., 1983), could solve these problems. Compared to 824 (pCASAAD), *Csh* thrives at a neutral pH, does not produce butanol or butyrate, and does not require antibiotics. It also uses an exceptionally broad spectrum of carbohydrate substrates similar to that of *Cac*.

**FIGURE 6:**
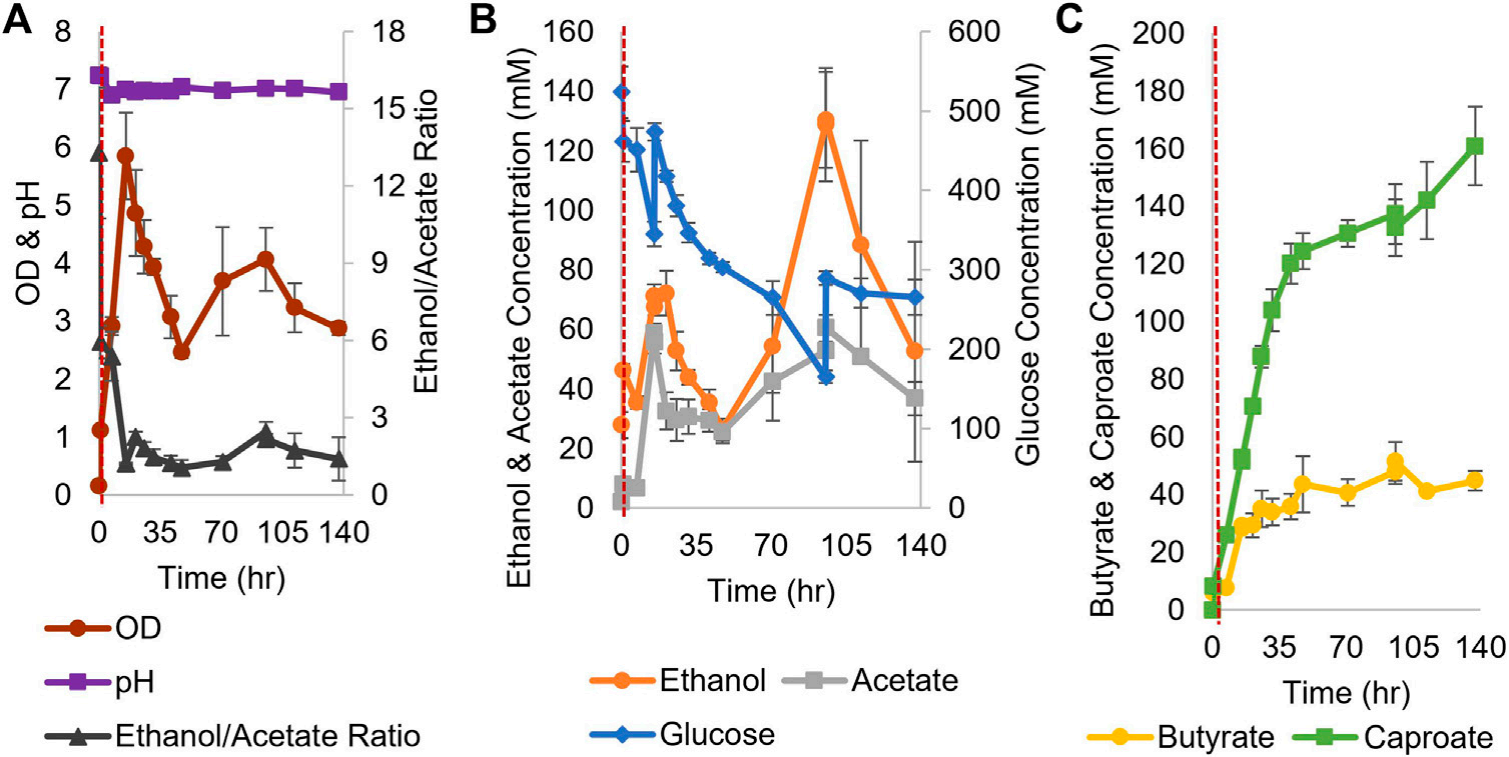
Growth and metabolite profiles of *Csh* and *Ckl* cocultures in pH-controlled bioreactors. **(A)** The OD_600_ and pH. The pH remained at its setpoint of 7 (*n* = 3 biological replicates). **(B)** shows the glucose profile and consumption (glucose was added twice; note the second axis) and the profiles of ethanol and acetate produced by *Csh*. **(C)** shows the profiles of the butyrate and caproate produced by *Ckl* in the coculture. Note that most caproate is produced in the initial 35 h, but that production continues until the end of the fermentation with an increase profile slope indicative of continued caproate production. The red dashed vertical line indicates *Ckl* inoculation.

For coculture experiments, *Csh* was inoculated first at an OD_600_ of 0.2 in the small bioreactors filled with anaerobic T-CGM-CC. After 30 min, concentrated *Ckl* cells were added at a 1:5 ratio to account for the lower growth rate of *Ckl*. The pH was maintained at 7 (Figure 6A). The ethanol/acetate ratio started high but was only maintained at around 1 (Figure 6B) due to the consumption of ethanol by *Ckl*. Glucose was added twice. *Ckl* almost immediately started producing caproate at a rate of 3.2 mM/h or 0.9 mM/h-OD_600_ (consistent with the other specific production rates in this work), and, importantly, maintained almost this rate of production for 40 h, producing 120 mM of caproate (Figure 6C). Even as low ethanol titers slowed the production rate to 0.3 mM/h, an average of 161 mM of caproate was produced before 140 h. Critically, these caproate titers are the highest published in any *Ckl* coculture than does not include *in situ* caproate removal.

These caproate production titers show that *Csh* is an exemplary coculture partner for *Ckl*. Since *Ckl* can produce over 200 mM of caproate in 45 h (Figure 2F), even higher coculture performance may be possible. Lowering the species ratio by adding additional *Csh* could have increased the ethanol titers and increased the ethanol to acetate ratio, leading to even faster performance (Figures 2C,D). *Csh* by itself produced ethanol to acetate in a ratio of around 3, but *Ckl* consumed more ethanol than acetate, keeping the ratio low. Nevertheless, the fast ethanol production of *Csh*, as opposed to the much slower ethanol production of *Cac*, led to this coculture’s greater success. While the peak ethanol titers from *Cac*-pCASAAD and *Csh* cocultures are similar, this does not account for all of the ethanol that was elongated into caproate by *Ckl* (in the *Csh* coculture, over 310 mM). Additionally, the compatible pH of *Csh* and *Ckl* led to a coculture without the negative effects of the autolysins and a more stable OD_600_. While no hexanol is produced, genetic modification of *Csh* or introduction of another coculture species could resolve this and further improve this novel coculture. Somewhat surprisingly, our efforts to identify heterologous fusion events in this *Csh-Ckl* coculture pair have not proved successful as yet. An essential difference between *Cac* and *Csh* is that they belong to different Clostridium-class genera, and that *Csh* is classified as Gram negative versus Gram positive for *Cac*.

## 4 Conclusion

Ethanologenic clostridia species are effective coculture partners for *Ckl*, and their fast production rates help to make carboxylic acid cocultures industrially viable. *Cac* cocultures outperform other published clostridia cocultures, and *Csh* delivers the highest-observed coculture caproate titers ever. *Cac* could be modified to increase ethanol titers further and to improve its pH tolerance, and *Csh* could be modified to produce even more ethanol or to produce alcohols from its carboxylic acids. *Clj* could also be added to capture lost carbon, and other strategies such as perfusion or biochar addition to capture the coculture products will increase productivity further.

## Data Availability

The raw data supporting the conclusion of this article will be made available by the authors, without undue reservation.
