# Supplementary material for: The potential of caproate (hexanoate) production using Clostridium kluyveri syntrophic cocultures with Clostridium acetobutylicum or Clostridium saccharolyticum
Source: Front Bioeng Biotechnol. 2022 Aug 22;10:965614. doi: 10.3389/fbioe.2022.965614 (PMC9441933; doi:10.3389/fbioe.2022.965614)
Supplement: Supplementary file 1 [file DataSheet1.PDF]

Supplementary Material

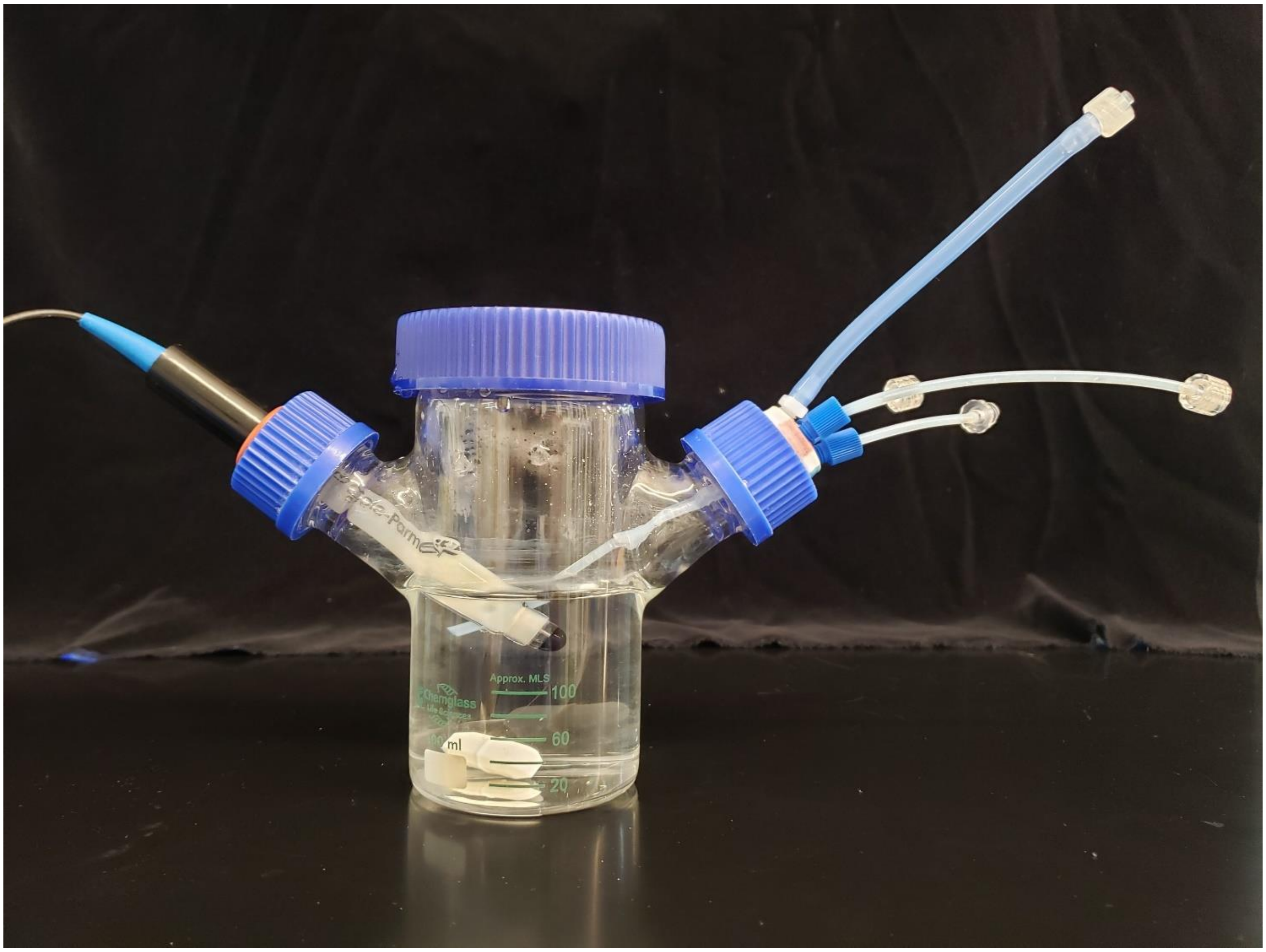

Supplementary Figure 1. An example of the spinner flasks used as miniature bioreactors. The central stirring axle is omitted in favor of a free-spinning bar. A pH probe is sealed into one sidearm with a grommet and the other sidearm holds the solvent delivery cap used for sampling, base addition, gas input, and gas exhaust.

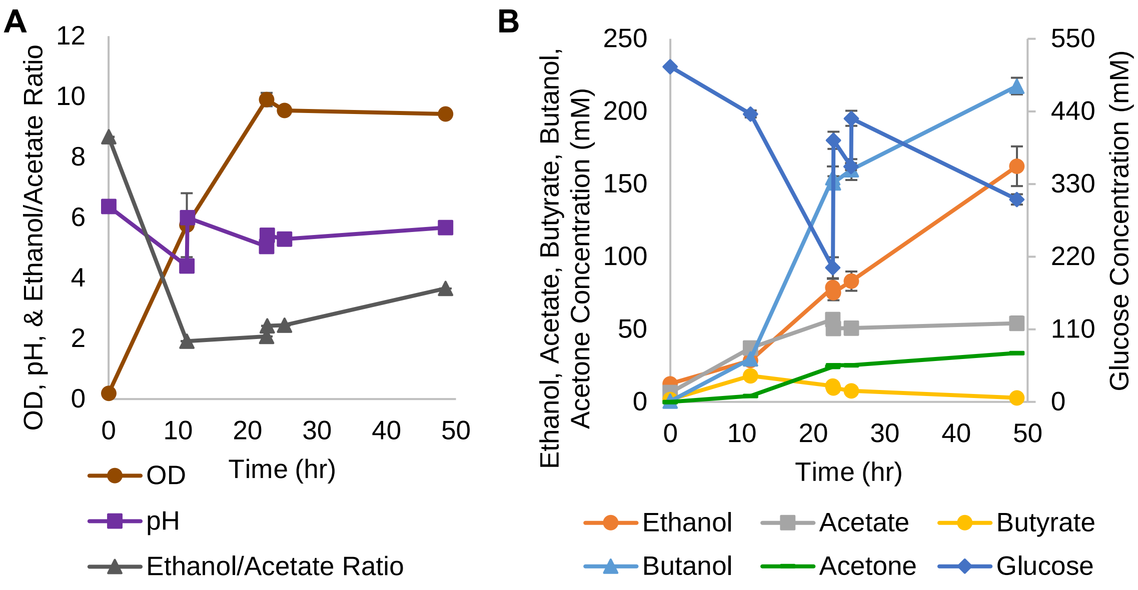

Supplementary Figure 2. Growth and metabolite profiles of *Cac*(pCASAAD) monocultures (n=3 biological replicates). With a pH maintained below 6, OD_{600} remains above 8 for 38 hours (A). Panel (B) shows glucose consumption (glucose was added twice; note the second axis) and product formation. 150 mM of ethanol was formed; both butyrate and acetone titers were low, but butanol titers exceeded 200 mM.
